# Gamete Therapeutics: Recombinant Protein Adsorption by Sperm for Increasing Fertility via Artificial Insemination

**DOI:** 10.1371/journal.pone.0065083

**Published:** 2013-06-10

**Authors:** Horacio Alvarez-Gallardo, Michael E. Kjelland, Juan F. Moreno, Thomas H. Welsh, Ronald D. Randel, Miguel A. Lammoglia, Mario Pérez-Martínez, Alma V. Lara-Sagahón, A. Enrique Esperón-Sumano, Salvador Romo

**Affiliations:** 1 Centro Nacional de Recursos Genéticos - Instituto Nacional de Investigaciones Forestales, Agrícolas y Pecuarias, Tepatitlán de Morelos, Jalisco, México; 2 Wayne State College, Wayne, Nebraska, United States of America; 3 Sexing Technologies, Navasota, Texas, United States of America; 4 Texas A&M University, College Station, Texas, United States of America; 5 Texas A&M AgriLife Research-Overton, Overton, Texas, United States of America; 6 Facultad de Ciencias Biológicas y Agropecuarias, Universidad Veracruzana, Tuxpan, Veracruz, México; 7 Facultad de Medicina Veterinaria y Zootecnia, Universidad Nacional Autónoma de México, México, Distrito Federal, México; 8 Facultad de Estudios Superiores Cuautitlán, Universidad Nacional Autónoma de México, Cuautitlán, Estado de México, México; Federal University of Parana (UFPR) ) – Campus Palotina, Brazil

## Abstract

A decrease in fertility can have a negative economic impact, both locally and over a broader geographical scope, and this is especially the case with regard to the cattle industry. Therefore, much interest exists in evaluating proteins that might be able to increase the fertility of sperm. Heparin binding proteins (HBPs), specifically the fertility associated antigen (FAA) and the Type-2 tissue inhibitor of metalloproteinase (TIMP-2), act to favor the capacitation and acrosome reaction and perhaps even modulate the immune system’s response toward the sperm. The objective of this research was to determine the effect on fertility of adding recombinant FAA (*r*FAA) and recombinant TIMP-2 (*r*TIMP-2) to bovine semen before cryopreservation for use in an artificial insemination (AI) program in a tropical environment. For this experiment, 100 crossbred (*Bos taurus* x *Bos indicus*) heifers were selected based on their estrus cycle, body condition score (BCS), of 4 to 6 on a scale of 1 to 9, and adequate anatomical conformation evaluated by pelvic and genital (normal) measurements. Heifers were synchronized using estradiol benzoate (EB), Celosil® (PGF2α) (Shering-Plough) and a controlled internal drug release (CIDR) device was inserted that contained progesterone. Inseminations were performed in two groups at random, 50 animals per group. The control group was inseminated with conventional semen. The treatment group was inseminated with semen containing *r*FAA (25 µg/mL) and *r*TIMP-2 (25 µg/mL). In the control group a 16% pregnancy rate was obtained versus a 40% pregnancy rate for the HBP treatment group, resulting in a significant difference (P = 0.0037). Given the results herein, one may conclude that the HBPs can increase fertility and could be an option for cattle in tropical conditions; however, one needs to consider the environment, nutrition, and the genetic interaction affecting the final result in whatever reproductive program that is implemented.

## Introduction

When the fertility decreases in males, it can present a negative economic impact on, among other things, the production of milk and meat from cattle [1]. The assisted reproduction technique (ART) of artificial insemination (AI) has played a central role in the genetic improvement of livestock. The general use of this technique, AI, and the success of its overall potential requires the preservation of semen for long periods of time. Even though cryopreservation helps with the prolonged storage of semen, the process of cryopreservation and thawing may cause irreversible damage to sperm [2]. Evidence suggests that the seminal plasma, which is a mixture of secretions originating in the testicles, epididymides, and accessory glands, contains molecules that affect the ability of the sperm to fertilize the oocyte [3]. These studies are based on the comparison of the seminal plasma composition of males with different levels of fertility, or by the identification of factors of the seminal plasma that facilitate or inhibit the sperm capacitation, fertilization, or related events [4].

Currently, many diverse proteins are being tested that may increase the fertilization capability of sperm, being that they act upon some process previous to pregnancy, and this is the case with the heparin binding proteins (HBPs), specifically the fertility associated antigen (FAA) and the Type-2 tissue inhibitor of metalloproteinase (TIMP-2). According to the scientific literature, HBPs have been viewed as actually favoring capacitation, acrosome reaction, and altering the immune system response toward the sperm.

### Heparin Binding Proteins

Heparin is chemically similar to the glycosaminoglycans (GAGs) that are secreted in the reproductive tract of the female. The heparin and the GAGs of the female reproductive tract bind to proteins on the surface of bovine sperm and this binding induces capacitation [5]. Evidence indicates that bulls exhibiting higher fertility produce ejaculates with more heparin binding protein on spermatozoal membranes, i.e., sperm with a higher binding affinity of heparin [6], compared to bulls with lower fertility [7]. It has also been reported that the sperm of bulls with a higher fertility have a larger frequency of acrosomal reaction in response to compounds similar to heparin and have a higher binding affinity by the heparin than those sperm from lower fertility bulls [8]. The binding of heparin by sperm has been documented in rabbits, monkeys, pigs, goats and humans [5].

The HBPs are produced by the accessory glands of the male and secreted within the seminal fluid. The accessory glands of the male rat (seminal vesicles, prostate, and Cowper’s gland) produce HBPs under the control of androgens [9]. The HBPs bind to the epididymal sperm and increase the ability of the acrosome reaction in response to the heparin and to the proteins of the zona pellucida [10].

The HBPs combine to form complexes with 5 levels of binding affinity for heparin. The complex with the greatest affinity for heparin, i.e., HBP-B5 is composed of multiple proteins (31, 24 and 14–18 kDa) [5]. Within the aforementioned group, one finds the 31 kDa HBP called FAA and the 24 kDa HBP identified as TIMP-2 [1], [11]. The presence of FAA (identified as a DNase-1) has been shown to be an indicator of superior fertility of bulls based on artificial insemination field fertility trials [7]. FAA has been identified in seminal vesicles and prostate, but has been detected very few times in bulbourethral glands [11].

The recombinant protein FAA has DNase-1 activity, and this can act on the neutrophil extracellular traps (NETs) in a similar manner to that of the natural protein in the seminal plasma [12]. Alghamdi et al. [13] found that the seminal plasma contained a protein factor or factors that can reduce the binding of neutrophils to the sperm *in vitro* in a dose dependent response. Based on what Alghamdi and Foster [12] have demonstrated, the activity of the DNase (FAA) present in the seminal plasma digests the exposed DNA and frees the trapped sperm, possibly resulting in more sperm reaching the oviduct and as such increasing fertility.

With respect to TIMP-2, it has been found in seminal vesicles, prostate, and bulboeurtheral glands. TIMP-2 acts to inhibit the enzymatic activity of the metalloproteases (MMPs), of which are included the interstitial collagens, gelatinases, estromelisines and membrane MMPs. Moreover, it has been found that TIMP-2 binds to the posterior region of the acrosome [14] [15].

Preliminary studies with the recombinant proteins indicate that in some cases they may help stabilize the acrosome membrane of the bovine sperm, determined by decreased post-thaw acrosome damage when FAA is added before cryopreservation. Barrios et al. [16] demonstrated that the absorption of isolated proteins from the seminal plasma can reduce the damage that cold shock can have on the sperm membrane. Results of a study by Mogielnicka-Brzozowska et al. [17] indicated that zinc-binding proteins of boar seminal plasma have a shielding effect on the plasma membrane and acrosome of spermatozoa, protecting these structures against consequences of cold shock. Mogielnicka-Brzozowska and Kordan [18] mentioned the possibility of adding specific plasma proteins to sperm for the rentention of features responsible for the efficient fertilization after storage.

The production of recombinant TIMP-2 and recombinant FAA has been described previously in the scientific literature. To obtain recombinant TIMP-2, an isolation of the total RNA was required from the bovine seminal vesicles, after which RT-PCR was used for the cloning, detection and the analysis of the sequence of the product obtained [19]. As a result, a fragment of 650 base pairs of the bovine TIMP-2 gene was obtained, which corresponds to 96% of the complete gene and contains all of the codons of the amino acid sequence for the desired TIMP-2 protein [19]. To obtain the FAA, a vector of highly induced expression was cloned in *E. coli*, a fragment of 592 base pairs from cDNA of FAA, corresponding to the amino acid residues 73 to 269 of the original protein [20].

The use of recombinant proteins for fertility trials in cattle is rare in the scientific literature. A recent *in vitro* fertilization (IVF) study by Ordonez-Leon et al. (2011) [21] and an AI study by Agado et al. (2011) [22] used recombinant FAA and TIMP-2 with frozen-thawed sex-sorted semen, but the results did not demonstrate a significant increase in fertility in either study. In another study, Amann et al. (1999) [23] used a synthetic peptide from rat prosaposin for a fertility field trial using cattle that resulted in improved fertility.

The present study aimed to test newer methods for increasing the fertility of cattle maintained in humid tropical conditions, more specifically, using the addition of recombinant heparin binding proteins (*r*HBPs) to conventional semen pre-cryopreservation. In this particular study, it appears that the difficult environmental conditions experienced by the cattle negatively impacted the results, as both control and treatment groups demonstrated a lower fertility than would be expected. However, the overall aim of demonstrating the applicability of these *r*HBPs was achieved, as the treatment group maintained a significantly higher fertility than the control group.

## Results

Compared to the control group, a larger proportion of gestations occurred in the animals with the HBP treatment, and for both body condition score (BCS) groups (Cochran-Maentel-Haenszel test, P = 0.0009). The frequencies and percentages of gestations observed are included in Table 1. The estimated homogeneous odds ratio was 8.37, indicating a strong association between the proportion of gestations and treatment with *r*HBPs for both BCS groups. Evidence that the effect of the *r*HBPs depended on body condition (Breslow-Day statistical test using the Tarone adjustment, P = 0.33) was not observed. The results of these tests indicate that a good model for explaining the proportion of the gestations is the logistic model with the effects for the *r*HBPs and the BCS [24]. A summary of the logistic regression analysis results are presented in Table 1 and Table 2 and show the percentages of gestations for the different experimental conditions estimated by the respective model using a 95% confidence interval.

**Table 1:** Comparison of body condition scores (BCS) and the number of gestational females in each experimental group which consisted of crossbred heifers inseminated with cryopreserved sperm with (treatment) or without (control) recombinant heparin binding proteins.

BCS	Groups	Number ofgestationalanimals	Total numberof animals	Percentage ofgestations	Percentage ofgestations*
4	Treatment	6	30	20	17.9 (8.3, 34.6)
	Control	0	22	0	2.8 (0.7, 10)
6	Treatment	14	20	70	73.0 (51.6, 87.3)
	Control	8	28	29	26.4 (13.8, 44.5)

* Estimated values with the logistic model (a 95% confidence interval).

**Table 2:** Fitted model of the logistic regression analysis of the pregnancy rates for estimating the percentages of gestations under the different experimental conditions: 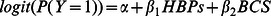.

Coefficients	Estimate	Std. Error	z-value	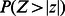
Intercept	−3.54	0.69	−5.14	2.7e-07
*r*HBPs	2.51	0.62	4.08	4.54e-05
BCS	2.02	0.60	3.40	0.0007

Null deviance: 30.63 on 3 degrees of freedom.

Residual deviance: 1.5 on 1 degree of freedom.

Akaike Information Criterion (AIC): 17.8.

*r*HBPs = recombinant heparin binding proteins, 0 or 1 for the treatment or control group, respectively.

BCS = body condition score, 0 or 1 for BCS = 4 or 6, respectively.

The percentage of controlled internal drug release (CIDR) devices retained was 100% for both the control group and the *r*HBP treatment group. Regarding the presentation of estrus, the control group experienced a 96% estrus and the *r*HBP group a 92% estrus; therefore not demonstrating a statistically significant difference between the groups (P = 0.19), presented in Table 3. The AI was done at 24 to 26 h after the second injection of estradiol benzoate (EB), or 48 to 50 h after the removal of the CIDRs in the animals that did not show signs of estrus.

**Table 3:** Percentages of controlled internal drug release (CIDR) devices retained, presentation of estrus and gestations summarized for the different groups.

	Number ofanimals	CIDRsretained	Presentation of estrus(P = 0.19)	Gestations(P = 0.0037)
Group A (Control)	50	50 (100%)	48 (96%)	8 (16%)
Group B (*r*HBPs)	50	50 (100%)	46 (92%)	20 (40%)
Total	100	100 (100%)	94 (94%)	28 (28%)

Group A (Control) = crossbred heifers inseminated with cryopreserved, frozen-thawed sperm without added recombinant heparin binding proteins (*r*HBPs), i.e., control group. Group B (*r*HBPs) = crossbred heifers inseminated with cryopreserved, frozen-thawed sperm with added *r*HBPs, i.e., treatment group.

### Natural FAA and TIMP-2 Levels Versus rFAA and rTIMP-2 Binding

**Figure 1:**
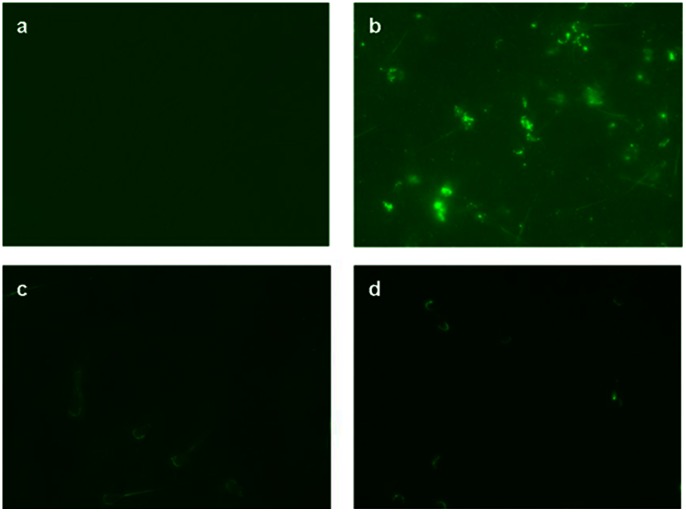
Visualization of representative control and treatment sperm samples included the following: a) fertility associated antigen (FAA) antibody control sample (FAA negative) without FAA present on sperm (provided by Midland Bioproducts Corporation®) and verified after the application of fluorescein conjugated FAA antibody, b) FAA protein control sample (FAA negative, provided by Midland Bioproducts Corporation®) with fluorescein conjugated FAA recombinant protein added to sperm, c) fluorescein conjugated Type-2 tissue inhibitor of metalloproteinase (TIMP-2) antibody added to sperm, and d) fluorescein conjugated TIMP-2 protein added to sperm.

**Figure 2:**
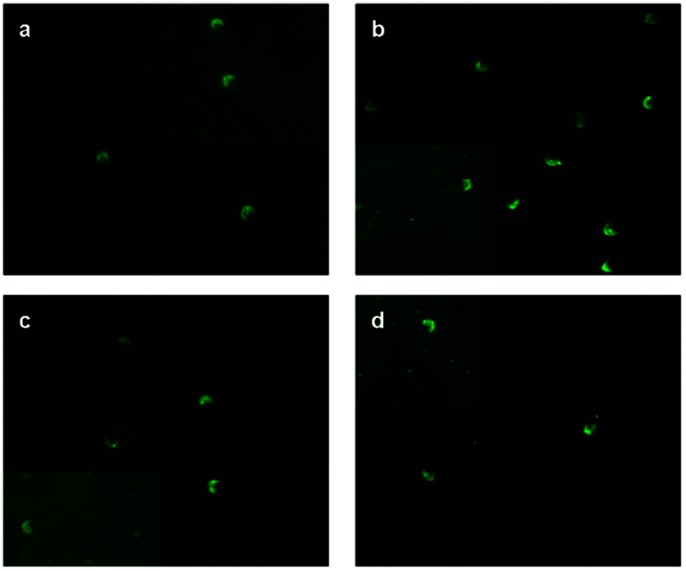
Sperm from the Brahman bull used in this study with: a) fluorescein conjugated Type-2 tissue inhibitor of metalloproteinase (TIMP-2) antibody revealing natural levels and location of TIMP-2 protein, b) fluorescein conjugated TIMP-2 recombinant protein adsorption by sperm, c) fluorescein conjugated fertility associated antigen (FAA) antibody revealing natural levels and location of FAA protein, and d) fluorescein conjugated FAA recombinant protein adsorption by sperm.

Bovine sperm samples testing negative for FAA, negative controls, from Midland Bioproducts Corporation® verified that the FAA conjugated antibody was not cross-reacting (Figure 1). Similar to McCauley et al. [11], depending upon the presence of either protein on sperm, the fluorescence binding patterns were observed in either the acrosomal region, postacrosomal region, acrosomal and postacrosomal regions, or no fluorescence if both proteins were absent (Figure 2).

## Discussion

The *r*HBP treatment group in the present research was 24% more fertile (based on pregnancy rate) than the control group, which is higher than that reported by Sprott et al. [7] who obtained an increase of 16.3% in the pregnancy rate in heifers with synchronized estrus, upon using frozen-thawed semen from FAA positive bulls. Notably, they obtained a 62% pregnancy rate and in the present research the results were lower, i.e., a 40% pregnancy rate. This difference in these results could be because in their study they used bulls that tested positive for only naturally occurring FAA and in the present research recombinant FAA and TIMP-2 were added, both produced via genetic engineering. In the present study, the overall pregnancy rate in the first service was 28%. This result is similar to that reported by Bó et al. [25] who reported pregnancy rates of 28.7% to 66.9% in cows and heifers treated with CIDRs, EB and fixed time artificial insemination (FTAI) 26–32 hours after CIDR removal.

During the course of this experiment, above normal ambient temperatures were prevalent and at times exceeded 40°C, which could have had an influence on the lower pregnancy percentages obtained. Another important aspect to consider is that during the hot period, the embryo death rate markedly increased during days 7 and 14 of gestation [26]. In terms of the molecular aspects related to the resistance of heat stress, one can find that heat shock proteins (HSP) have been detected not only in gametes but also in early stage embryos. The HSP can increase or decrease the expression of the different isoforms of the progesterone receptors, which in turn can increase or decrease the effect of progesterone; this aspect can influence the levels of progesterone, and therefore can influence the gestation [27].

Since this experiment was conducted during the dry season, there was not an abundance of forage and the available forage was not of good nutritional quality. This aspect of available forage affects the metabolism of the individuals and generates a negative energy balance (NEB). It is well documented that insulin-like growth factor 1 (IGF-1) increases the number of binding sites to luteinizing hormone (LH) and potentially the production of LH. The NEB causes a decrease in the circulating concentration of IGF binding proteins (IGFBPs). This decrease in IGFBPs can affect the luteinization of the corpus luteum (CL), which could generate a dysfunctional CL, possibly resulting in the insufficient production of progesterone for maintaining the gestation and ultimately could cause the resorption of the embryo [28].

In terms of the results obtained for the AI at first service utilizing the two types of semen, the fertility of the females inseminated with the *r*HBPs (FAA and TIMP-2) added to the semen increased compared to the control group. Given these aforementioned results, one can conclude that the addition of the *r*HBPs utilized in this study can improve fertility, consequently increasing the pregnancy rate in heifers. In this way, the addition of these proteins to bovine semen before cryopreservation can be a useful tool to improve the results of reproductive programs that involve cattle in tropical climates. With regard to the pregnancy rate in relation to BCS, some heifers with a poor body condition presented gestation with the *r*HBPs, while not a single one succeeded in producing a gestation with the control semen. Based on this, it is possible to conclude that the addition of *r*HBPs to the semen can increase fertility in cattle when conditions may not be favorable for reproduction under normal circumstances. Until now, this is the first research effort that reports the successful use of *r*FAA and *r*TIMP-2 in a synchronized AI program in cattle under tropical conditions.

Under the previously mentioned conditions, the HBP treated semen increased the fertility with respect to the control group. Although we used cattle as a model in the present research, the application could be extended for wider use in domestic animal husbandry, assisted reproductive techniques for endangered species, and even human clinical treatments. We recommend that additional experiments be conducted to determine if the positive results in the present study can be achieved under different environmental conditions, other breeds of cattle, or even in other species.

With advances in such fields as proteomics it is now possible to identify proteins much more efficiently. Similarly, given the increasing efficiency in recombinant production techniques, there will likely become a more widespread use as the costs of production decrease. Although our study is limited in terms of sample size, due to the expense of the recombinant proteins, this investigation does demonstrate that the potential use for fertility enhancement applications exists. Perhaps this work will provoke other creative ideas or inspire others to consider alternative uses for such biotechnologies. For example, if recombinant proteins can act to deter neutrophils, if that is what is truly occurring based on previous research; then perhaps similar deterrent or cloaking mechanisms could be designed for other situations.

## Materials and Methods

### Ethics Statement

All of the animals used in this research were treated in accordance to the Federation of Animal Science Societies (FASS) guide for use of farm animals in research and teaching [29]. The present study took place between May 16 and July 3, 2010 in the state of Tabasco, Mexico, situated in the humid tropical region of Mexico, Latitude North 17°59′ and Latitude West 92°56′ at an elevation of 10 m above sea level. The climate is typically hot and humid with abundant rainfall in the summer with an average temperature in this region of ∼33.6°C and annual cumulative rainfall of 2,237 mm [30].

### Semen Collection and Semen Characteristics

Semen was collected from one Brahman bull by means of an artificial vagina. The ejaculates were only used for processing conventional straws of semen if they met the following criteria: minimum motility of ≥55%, primary abnormal morphologies ≤15%, secondary abnormal morphologies ≤15%, and a total abnormal morphology count not to exceed 25%. Further, samples used in the post-thaw analyses had to meet standard quality control conditions of motility ≥45% at 0 h and ≥30% at 3 h when incubated at 37°C. The post-thaw intact acrosomes were ≥50% at 3 h.

For all semen quality evaluations, 25.4 × 76.2 mm glass microscope slides (Andwin, Addison, IL) and 22 × 22 mm #1.5 coverslips (Thomas Scientific, Swedesboro, NJ, USA) were used. All motility assessments were made using brightfield microscopy and post-thaw intact acrosomes and morphology assessments utilized differential interference contrast (DIC) microscopy at 400X. Samples were viewed using a Nikon Eclipse 80*i* microscope (Nikon Instruments Inc., Melville, NY, USA).

### Obtaining Recombinant FAA and Recombinant TIMP-2

The present study formed part of a larger collaborative project between Texas A&M University, Sexing Technologies, and the Facultad de Estudios Superiores Cuautitlan, UNAM. The semen, both with and without the HBPs, was prepared and cryopreserved by Sexing Technologies. The recombinant FAA and TIMP-2 proteins used in the present study were provided by TMI Laboratories International, Inc. and added at Sexing Technologies.

### Identification of Natural Levels of FAA and TIMP-2: Indirect Immunofluorescence

TIMP-2 antibody (ab38975) (Abcam Inc., Cambridge, MA, USA) and FAA antibody (Midland Bioproducts Corporation®) were purchased and directly conjugated using Lightning-Link™ Fluorescein conjugation kit (Innova Biosciences: Product # 707 - 0010, 0015, Batch # 606, 678, and 686). VECTASHIELD® Mounting Medium for Fluorescence (Cat. No. H-1000, Lot# U1017, Vector Laboratories Inc., Burlingame, CA, USA) was used to reduce photobleaching. Samples were viewed using a Nikon Eclipse 80*i* microscope with fluorescence capabilities and NIS Elements BR 3.00 imaging software [31] with a CCD camera, specifically a Photometrics® CoolSNAP™ 164 *EZ* (Photometrics, Tucson, AZ, USA), for photographing.

To verify that the FAA conjugated antibody was not cross-reacting, negative controls were used. Bovine sperm samples that tested negative for FAA, i.e., sperm not having FAA protein, were obtained from Midland Bioproducts Corporation®.

### Addition and Verification of FAA and TIMP-2 Binding

For this research, 25 µg of FAA and 25 µg of TIMP-2 were added per milliliter of semen, and then left to adsorb for 20 min at ∼21°C, after which the semen was diluted and cooled to about 5°C prior to packaging and cryopreservation. Adsorption of the recombinant proteins was verified both by directly conjugating the recombinant proteins using the Lightning-Link™ Fluorescein conjugation kit (Innova Biosciences: Product # 707 - 0010, 0015, Batch # 606, 678, and 686), as well as using the conjugated antibody procedure similar to that used in identifying the natural levels of FAA and TIMP-2. Samples were viewed both before and after treatment with recombinant proteins using a Nikon Eclipse 80*i* microscope with fluorescence capabilities and NIS Elements BR 3.00 imaging software [31] with a CCD camera, specifically a Photometrics® CoolSNAP™ 164 *EZ* (Photometrics, Tucson, AZ, USA), for photographing.

### Semen Extender Preparation

All of the chemicals used in making the semen extenders were purchased from Amresco (AMRESCO® Inc., Solon, OH, USA). The semen extenders used in the experiments were prepared using the standard protocols and procedures within Sexing Technologies and consisted of the same formulation having a pH of 6.8 and an osmolality balanced at ∼300±5 mOsm/Kg for the Tris A extender. For testing on a batch-to-batch basis, Tris B extender was diluted 3∶1 (A:B) and vortexed with a final osmolality of 830 mOsm/Kg [32]. The formula of the Tris A semen extender utilized consisted of the following: 7.57 g TRIS, 4.32 g Citric Acid Monohydrate (CAM), 2.25 g Fructose, 50 mL (20% v/v) egg yolk, 50 µg/mL Tylosin, 250 µg/mL Gentamycin, 150/300 µg/mL Lincomycin-Spectinomycin, and Nanopure Water 18.2 MilliQ added to make a total of 250 mL extender. The antibiotic guidelines are approved by Certified Semen Services of the National Association of Animal Breeders (NAAB). The Tris B extender differs from the Tris A extender in that it contains glycerol, i.e., 14% (v/v). The Tris A extended fraction containing the semen sample is allowed to cool to about 5°C, after which the Tris B extender of similar temperature is added incrementally, in order to reduce osmotic shock. The final mix of the two extenders used to dilute the bull semen pre-cryopreservation was a 1∶1 ratio of A and B fractions of respective extenders, with a final glycerol content of 7%. Semen was placed in 0.5 mL french straws at a concentration of 25 × 10^6^ sperm per straw (IMV Technologies, L’Aigle, France) and cryopreserved using an automated freezing device, IMV Digitcool® (IMV, Cedex, France) and stored in liquid nitrogen.

### AI of Heifers

For this experiment, a total of 100 tropically-adapted cross-bred commercial heifers (*Bos indicus* × *Bos taurus* genotype) were selected. From these animals, two groups were formed by random selection; the heifers in the control group (n = 50) were inseminated with semen not having *r*HBPs added and the treatment group heifers (n = 50) were inseminated with semen having the added *r*HBPs (*r*FAA and *r*TIMP-2).

### Inclusion Criteria

The heifers were selected for this experiment by taking into account their cyclicity, BCS, weight, anatomic conformation and genital anatomy.

#### Cyclicity

The ovarian activity was evaluated by means of real-time ultrasound (mode-B) with a 7.5 MHz linear array transducer (VetPreg, China) and the selected animals had at least a follicle size of ≥8 mm and a CL.

#### Body condition and anatomic conformation

Animals were selected with a BCS of 4 (average of 320 kg) to 6 (average of 350 kg) on a scale of 1 to 9 where 1 is very thin and 9 is considered obese [33]. The anatomical conformation was evaluated by means of pelvimetry [34].

#### Genital anatomy

Animals were selected with normal genital anatomy with reference to the presence of ovaries, uterine horns, cervix and vulva, by means of transrectal palpation.

#### Exclusion criteria

Females with anatomic abnormalities, anestrus and/or with BCS of 3 or less were not selected for the experiment.

### Estrus Synchronization and Ovulation

The heifers were synchronized for presenting estrus in the following manner: Day 0, they received an intramuscular (IM) injection of 2 mg of EB, 500 µg of Celosil® prostaglandin F2α (PGF2α; Schering-Plough, México) and a CIDR was inserted containing 1.38 g of progesterone; Day 7, the CIDR was removed and heifers were administered 500 µg of Celosil® (PGF2α) by IM injection; Day 8, heifers were IM injected with 0.5 mg EB [25], [35].

### Estrus Detection

Direct observation was the method used for detecting estrus in the heifers, and once identified the base of the tail was painted with a colored marker. The marking was done on Day 8 of synchronization at the time of injecting the second dose of EB. The observation for detection of estrus began at 6∶00 to 7∶00 pm on Day 8 of synchronization and at 5∶00 am on Day 9 of synchronization. Those heifers that lost their colored marker were inseminated first [36], given that in a tropical climate the presentation of estrus typically occurs between 6∶00 pm and 6∶00 am [37]. The animals that were not identified as being in estrus were inseminated at a fixed time of 52 to 56 h after the removal of the CIDR.

### Artificial Insemination

The heifers were inseminated using the rectovaginal technique and forming 2 groups at random of 50 animals each. The control group was inseminated with cryopreserved bovine semen without HBPs and the HBP treatment group was inseminated with cryopreserved bovine semen containing 25 µg/mL of FAA and 25 µg/mL TIMP-2. The inseminations were performed 24 to 26 h after the second injection of EB and removal of the CIDR.

### Pregnancy Diagnosis

Pregnancy was diagnosed by means of real-time ultrasound (mode-B) with a linear array transducer of 7.5 MHz (VetPreg, China), at 45 days post insemination.

### Statistical Analysis

In both groups, treatment and control, the percentage of gestation at first service was determined, as well as gestation correlating to body condition. The Breslow-Day statistical test using the Tarone adjustment [38] was utilized to test the interaction effects of the BCS and the *r*HBPs on the gestation rate. The independence of the gestation rate and the treatment with *r*HBPs factoring for the BCS was tested using the Cochran-Mantel-Haenszel test. A logistic model was also created with the effects of the *r*HBPs and the BCS for estimating the percentages of gestation and associated standard error. The statistical analysis was made with the software R [39]; specifically for the Breslow-Day test the metafor package of R [40] was utilized. A comparison was made of the proportions of the animals that entered estrus in each group by means of a Z test.

## References

[1] McCauley TC, Zhang HM, Bellin ME, Ax RL (2001) Identification of a heparin-binding protein in bovine seminal fluid as tissue inhibitor of metalloproteinases-2. Mol Reprod Dev 58: 336–341. 11170275 10.1002/1098-2795(200103)58:3<336::AID-MRD12>3.0.CO;2-Z

[2] Harshan HM, Singh LP, Arangasamy A, Ansari MR, Kumar S (2006) Effect of buffalo seminal plasma heparin binding protein (HBP) on freezability and in vitro fertility of buffalo cauda spermatozoa. Anim Reprod Sci 93: 124–133. 16143473 10.1016/j.anireprosci.2005.07.010

[3] Nauc V, Manjunath P (2000) Radioimmunoassays for bull seminal plasma proteins (BSP-A1/−A2, BSP-A3, and BSP 30 kilodaltons), and their quantification in seminal plasma and sperm. Biol Reprod 63: 1058–1066. 10993827 10.1095/biolreprod63.4.1058

[4] Killian GJ, Chapman DA, Rogowski LA (1993) Fertility-associated proteins in Holstein bull seminal plasma. Biol Reprod 49: 1202–1207. 8286602 10.1095/biolreprod49.6.1202

[5] Miller DJ, Winer MA, Ax RL (1990) Heparin-binding proteins from seminal plasma bind to bovine spermatozoa and modulate capacitation by heparin. Biol Reprod 42: 899–915. 2383614 10.1095/biolreprod42.6.899

[6] Marks JL, Ax RL (1985) Relationship of nonreturn rates of dairy bulls to binding affinity of heparin to sperm. J Dairy Sci 68: 2078–2082. 4044971 10.3168/jds.S0022-0302(85)81070-5

[7] Sprott LR, Harris MD, Forrest DW, Young J, Zhang HM, et al (2000) Artificial insemination outcomes in beef females using bovine sperm with a detectable fertility associated antigen. J Anim Sci 78: 795–798. 10784166 10.2527/2000.784795x

[8] Bellin ME, Hawkins HE, Ax RL (1994) Fertility of range beef bulls grouped according to presence or absence of heparin-binding proteins in sperm membranes and seminal fluid. J Anim Sci 72: 2441–2448. 8002463 10.2527/1994.7292441x

[9] Nass SJ, Miller DJ, Winer MA, Ax RL (1990) Male accesory sex glands produce heparin-binding proteins that bind to cauda epididymal spermatozoa and are testosterone dependent. Mol Reprod Dev 25: 237–246. 2331373 10.1002/mrd.1080250305

[10] Ax RL, Hawkins HE, DeNise SK, Holm TR, Zhang HM, et al.. (2002) New developments in managing the bull. In: Fields MJ, Sand RS, Yelich JV, editors. Factors Affecting calf crop: biotechnology of reproduction. CRC Press LLC, Florida, USA. 287–295.

[11] McCauley TC, Zhang HM, Bellin ME, Ax RL (1999) Purification and characterization of fertility-associated antigen (FAA) in bovine seminal fluid. Mol Reprod Dev 54: 145–153. 10471474 10.1002/(SICI)1098-2795(199910)54:2<145::AID-MRD6>3.0.CO;2-6

[12] Alghamdi AS, Foster DN (2005) Seminal DNase frees spermatozoa entangled in neutrophil extracelular traps. Biol Reprod 73: 1174–1181. 16107606 10.1095/biolreprod.105.045666

[13] Alghamdi AS, Foster DN, Troedsson MH (2004) Equine seminal plasma reduces sperm binding to polymorphonuclear neutrophils (PMNs) and improves the fertility of fresh semen inseminated into inflamed uteri. Reproduction 127: 593–600. 15129015 10.1530/rep.1.00096

[14] McCauley TC, Bellin ME, Ax RL (1996) Localization of a heparin-binding protein to distinct regions of bovine sperm. J Anim Sci 74: 429–438. 8690680 10.2527/1996.742429x

[15] Dawson GR (2005) Localization on sperm, quantification and molecular features of two seminal proteins. PhD Dissertation, Arizona University, USA. 164 p.

[16] Barrios B, Pérez-Pérez R, Gallego M, Tato A, Osada J, et al (2000) Seminal Plasma Proteins Revert the Cold-Shock Damage on Ram Sperm Membrane. Biol Reprod 63: 1531–1537. 11058562 10.1095/biolreprod63.5.1531

[17] Mogielnicka-Brzozowska M, Wysocki P, Strzezek J, Kordan W (2011) Zinc-binding proteins from boar seminal plasma – isolation, biochemical characteristics and influence on spermatozoa stored at 4°C. Acta Biochim Pol 58: 171–177. 21584285

[18] Mogielnicka-Brzozowska M, Kordan W (2011) Characteristics of selected seminal plasma proteins and their application in the improvement of the reproductive processes in mammals. Pol J Vet Sci 14(3): 489–499. 21957748 10.2478/v10181-011-0074-z

[19] Zhang H, Bellin M, Ax R (2001) Cloning and expression of recombinant bovine tissue inhibitor. Plant & Animal Genome IX Conference, San Diego, CA, USA.

[20] Lenz RW, Zhang HM, Oyarzo JN, Bellin ME, Ax RL (2000) Bovine fertility-associated antigen (FAA) and a recombinant segment of FAA improve sperm function. Society for the Study of Reproduction, Abstract # 80.

[21] Ordonez-Leon EA, Kjelland ME, Moreno JF, Welsh TH, Randel RD, et al (2011) *In vitro* fertilization using frozen-thawed sexed semen treated with recombinant heparin-binding proteins. Reprod Fertil Dev 24(1): 197–198.

[22] Agado BJ, Neuendorff DA, Shafer GL, Kjelland ME, Moreno J, et al.. (2011) The influence of the addition of heparin binding protein and tissue inhibitors of metalloproteinases-2 to sexed bovine semen on conception rate and pregnancy rate. J Anim Sci Vol. 89, E-Suppl. 1/J. Dairy Sci. Vol. 94, E-Suppl. 1, p. 498.

[23] Amann RP, Seidel GE, Brink ZA (1999) Exposure of thawed frozen bull sperm to a synthetic peptide before artificial insemination increases fertility. J Androl 20: 42–46. 10100472

[24] Agresti A (2007) An introduction to categorical data analysis. Second Edition. John Wiley & Sons Inc. New Jersey. 400 p.

[25] Bó GA, Baruselli PS, Martínez MF (2003) Pattern and manipulation of follicular development in *Bos indicus* cattle. Anim Reprod Sci 78: 307–326. 12818651 10.1016/s0378-4320(03)00097-6

[26] Berg DK, Van Leeuwen J, Beaumont S, Berg M, Pfeiffer PL (2010) Embryo loss in cattle between Days 7 and 16 of pregnancy. Theriogenology 73: 250–260. 19880168 10.1016/j.theriogenology.2009.09.005

[27] Rekawiecki R, Kowalik MK, Slonina D, Kotwica J (2008) Regulation of progesterone synthesis and action in bovine corpus luteum. J Physiol Pharmacol 59 Suppl 975–89. 19261973

[28] Waterman RC, Butler WR (2010) Metabolic signals of the beef cow in negative energy balance. Proceedings, Grazing Livestock Nutrition Conference. 93–100.

[29] Federation of Animal Science Societies (2010) Guide for the care and use of agricultural animals in research and teaching, third edition. 169 p. Available: http://iacuc.ufl.edu/AnimalUseGuides/Ag_Guide_3rd_ed.pdf. Accessed 8 September 2012.

[30] Enciclopedia de los Municipios de México (2010) INAFED-SEGOB. Available: http://www.e-local.gob.mx/wb2/ELOCAL/ELOC_Enciclopedia. Accessed 15 September 2012.

[31] Nikon (2008) NIS Elements BR 3.00. Available: http://www.nis-elements.com. Accessed 8 September 2012.

[32] Lenz RW, Kjelland ME, Vonderhaar K, Swannack TM, Moreno JF (2011) A comparison of bovine seminal quality assessments using different viewing chambers with a computer-assisted semen analyzer. J Anim Sci 89(2): 383–388. 20952528 10.2527/jas.2010-3056

[33] Herd DB, Sprott LR (1986) Body condition, nutrition and reproduction of beef cows. Extension Bulletin. No 1526, Texas A&M University, College Station, Texas, USA. 12 p.

[34] Gene HD (1992) Pelvic measurements for reducing calving difficulty. Beef cattle handbook. University of Nebraska. USA. 7 p.

[35] Cutaia LE, Peres LC, Pincinato D, Chesta PM, Ramos M, et al.. (2010) Programas de sincronización de celos en vaquillonas de carne: Puntos críticos a tener en cuenta. Primer Curso de reproducción bovina Syntex. Villahermosa, Tabasco, México.

[36] Rae DO (2002) Bovine estrus: tools for detection and understanding. In: Fields MJ, Sand RS, Yelich JV, editors. Factors Affecting calf crop: biotechnology of reproduction. CRC Press LLC, Florida, USA. 7–20.

[37] Galina CS, Orihuela A (2007) The detection of estrus in cattle raised under tropical conditions: what we know and what we need to know. Horm Behav 52: 32–38. 17482614 10.1016/j.yhbeh.2007.03.025

[38] Tarone RE (1985) On heterogeneity tests based on efficient scores. Biometrika 72: 91–95.

[39] R Development Core Team (2012) R: A language and environment for statistical computing. Vienna, Austria. ISBN 3-900051-07-0. Available: http://www.R-project.org/. Accessed 8 September 2012.

[40] Viechtbauer W (2010) Conducting meta-analyses in R with the metafor package. J Stat Softw 36(3), 1–48. Available: http://www.jstatsoft.org/v36/i03/. Accessed 8 September 2012.

